# Dysphagia in Acute Stroke: Incidence, Burden and Impact on Clinical Outcome

**DOI:** 10.1371/journal.pone.0148424

**Published:** 2016-02-10

**Authors:** Marcel Arnold, Kai Liesirova, Anne Broeg-Morvay, Julia Meisterernst, Markus Schlager, Marie-Luise Mono, Marwan El-Koussy, Georg Kägi, Simon Jung, Hakan Sarikaya

**Affiliations:** 1 Department of Neurology, University Hospital of Berne, Berne, Switzerland; 2 Department of Neuroradiology, University Hospital of Berne, Berne, Switzerland; 3 Department of Neurology and Neurophysiology, Kantonsspital St. Gallen, St. Gallen, Switzerland; Weill Cornell Medical College, UNITED STATES

## Abstract

**Background:**

Reported frequency of post-stroke dysphagia in the literature is highly variable. In view of progress in stroke management, we aimed to assess the current burden of dysphagia in acute ischemic stroke.

**Methods:**

We studied 570 consecutive patients treated in a tertiary stroke center. Dysphagia was evaluated by using the Gugging Swallowing Screen (GUSS). We investigated the relationship of dysphagia with pneumonia, length of hospital stay and discharge destination and compared rates of favourable clinical outcome and mortality at 3 months between dysphagic patients and those without dysphagia.

**Results:**

Dysphagia was diagnosed in 118 of 570 (20.7%) patients and persisted in 60 (50.9%) at hospital discharge. Thirty-six (30.5%) patients needed nasogastric tube because of severe dysphagia. Stroke severity rather than infarct location was associated with dysphagia. Dysphagic patients suffered more frequently from pneumonia (23.1% vs. 1.1%, *p*<0.001), stayed longer at monitored stroke unit beds (4.4±2.8 vs. 2.7±2.4 days; *p*<0.001) and were less often discharged to home (19.5% vs. 63.7%, *p* = 0.001) as compared to those without dysphagia. At 3 months, dysphagic patients less often had a favourable outcome (35.7% vs. 69.7%; *p*<0.001), less often lived at home (38.8% vs. 76.5%; *p*<0.001), and more often had died (13.6% vs. 1.6%; *p*<0.001). Multivariate analyses identified dysphagia to be an independent predictor of discharge destination and institutionalization at 3 months, while severe dysphagia requiring tube placement was strongly associated with mortality.

**Conclusion:**

Dysphagia still affects a substantial portion of stroke patients and may have a large impact on clinical outcome, mortality and institutionalization.

## Introduction

Dysphagia is a common complication of stroke, but estimates of its frequency vary considerably [1,2]. It is an important cause of pneumonia within the first days after stroke and previous studies reported an increased risk of mortality in the acute phase [3–5]. Furthermore, dysphagia has been shown to be associated with malnutrition, dehydration and increased length of hospital stay [6]. Over the last decades, acute stroke management has substantially improved in many countries through reperfusion techniques, introduction of stroke units and stroke prevention. Accordingly, several studies have reported a substantial decline in both stroke incidence and associated mortality in developed countries [7–9]. Considering these advances in stroke management, we were interested in the current incidence and burden of dysphagia in a comprehensive stroke center and its association with pneumonia, discharge destination and clinical outcome at 3-month follow-up.

## Methods

The Inselspital is a tertiary stroke center and the main teaching hospital of the University of Bern, Switzerland, with a catchment area of approximately 2 million inhabitants. The Bernese Stroke Registry is a computer-based system and was set up in January 2000 to prospectively collect data from consecutive acute stroke patients at Inselspital. The study was approved by the Local Ethics Committee Bern. Patients with acute ischemic stroke admitted between January 2012 and November 2013 were retrospectively evaluated. The authors had access to identifying patient information. Data was anonymized prior to author access.

Demographic variables, vascular risk factors, infarct location and the use of thrombolysis were systematically recorded. Stroke territory was divided into anterior and posterior, based on previously used definitions [10,11]. Among strokes in posterior cerebral circulation, brainstem involvement was registered separately as it is supposed to be strongly associated with dysphagia. Stroke severity at admission was assessed with the National Institutes of Health Stroke Scale (NIHSS) score [12]. All patients treated with thrombolysis were admitted to a stroke unit for at least 24 hours. Clinical outcome at 3 months was evaluated by a certified stroke nurse or neurologist using the modified Rankin Scale (mRS) score [13]. The following parameters were also assessed: mortality during hospitalization and at 3 months, discharge destination and living situation at 3 months, length of hospital stay for different units (stroke unit vs. ward), incidences of dysphagia and pneumonia as well as the frequency of antibiotic treatment and nasogastric tube insertion. Pneumonia was diagnosed by the treating physician according to Centers for Disease Control and Prevention (CDC) criteria and based on typical findings in pulmonary examination (tachypnoea, inspiratory crackles, bronchial breathing) or chest radiograph (infiltration, consolidation), and additional evidence of either fever (body temperature >38°C), cough with purulent sputum or pathogen isolation in either blood or sputum culture [14]. Experienced physiotherapists with special training in dysphagia regularly checked the swallowing ability in each stroke patient within 24 hours after admission by using the Gugging Swallowing Screen (GUSS) and oral feeding was withheld until intact swallowing was demonstrated [15]. Dysphagia evaluation started with part 1 of GUSS, which provides one point for each of the following 5 criteria: (I) patient is vigilant, (II) normal voluntary coughing, (III) normal deglutition of saliva (IV) without drooling and (V) without voice change. If patients had a GUSS score <5 points in part 1, further dysphagia evaluation was performed by additional investigations (Videofluoroscopic Evaluation of Swallowing (VFES) or Fiberoptic Endoscopic Evalutation of Swallowing (FEES) or placement of a nasogastric tube, depending on the judgment of the treating therapist and physician. If the maximum of 5 points was achieved in part 1 of GUSS, the next subset (direct swallowing test, part 2) of GUSS was continued consisting of 3 subtests (semisolid swallowing, liquid swallowing, solid swallowing). The highest possible score in the GUSS is 20 points (5 points in part 1 and 15 points in part 2), denoting normal swallowing ability. For this study, dysphagia was defined as GUSS score of 19 points or less. A score of less than 10 points was defined as severe dysphagia with high aspiration risk, thus these patients received a nasogastric tube (“nil by mouth”) [15]. In patients with GUSS score between 10 and 19 points, stepwise special diet was ordered depending on severity of dysphagia [16].

The primary aim of this study was to assess the incidence of dysphagia in stroke patients and to compare the clinical outcomes at 3 months, namely death and favourable outcome (defined as mRS 0–1). In addition, we aimed to evaluate predictors of dysphagia and potential association with following secondary endpoints: occurrence of pneumonia, use of antibiotics and chest radiographs, length of hospital stay in various units, discharge destination and institutionalization at 3 months.

## Statistical Analysis

Normally distributed data were expressed as mean±standard deviation (SD) and compared using Students t-test. The 2 groups (patients with dysphagia vs. patients with normal swallowing ability) were compared using Mann Whitney U-test for continuous variables and the Pearson’s chi-square test for binary variables. Multivariate logistic regression analysis was performed to assess the independent effects of dysphagia and the other predictors on the outcome parameters favourable outcome, institutionalization and mortality at 3 months. In a first step, the influence of every single potential predictor on the outcomes was evaluated using univariate logistic regression analysis. The parameters examined were age, baseline NIHSS score, sex, infarct location (anterior vs. posterior cerebral circulation), brainstem infarction, arterial hypertension, hypercholesterolemia, diabetes and smoking. In a second step, a multivariate logistic regression analysis was performed, including all potential predictors with a *p*-value <0.2 from univariate analyses. Significance was set at *p*<0.05 level.

## Results

A total of 570 consecutive patients with ischemic stroke were included in this study. Mean age was 65.1 years (range, 19.6–94.7 years), 366 (64.2%) patients were male and thrombolysis was performed in 156 (27.3%) patients. Ischemic stroke affected the anterior cerebral circulation in 436 (76.5%) patients, posterior cerebral circulation in 104 (18.3%) and both territories in 28 (4.9%). Exact stroke localization could not be established in 2 patients. Brainstem infarction was present in 82 of 104 (78.9%) patients with posterior circulation stroke.

Dysphagia was diagnosed in 118 (20.7%) patients (mean age, 65.6; range, 23.5–91.0 years). The clinical characteristics of patients with and without dysphagia are shown in Table 1. Patients with dysphagia had more severe neurological deficits at baseline (mean NIHSS score 9.7±7.0 vs. 4.5±5.1; *p*<0.001) and had less often hypertension as compared to patients without dysphagia (36.2% vs. 63.7%; p = 0.029). Other clinical characteristics were similar in both groups.

**Table 1:** Baseline characteristics.

	No dysphagia (n = 452)		Dysphagia (n = 118)		*p*
Age	64.9±14.0		65.6±14.5		0.671
NIHSS at baseline	4.5±5.2		9.8±7.0		0.000
Male gender	63.7%	288/452	66.1%	78/118	0.630
Arterial hypertension	63.7%	288/448	36.2%	42/116	0.029
Hypercholesterolaemia	66.1%	291/440	61.9%	73/118	0.387
Diabetes mellitus	19.1%	84/441	21.2%	25/118	0.602
Smoking	31.5%	130/413	38.7%	41/106	0.159
Anterior stroke territory	75.4%	341/452	80.5%	95/118	0.321
Posterior stroke territory	19.0%	86/452	15.3%	18/118	0.345
Anterior and posterior stroke territory	5.1%	23/452	4.2%	5/118	1.000
Brainstem involvement	13.7%	62/452	17.0%	20/118	0.373
Thrombolysis	19.9%	90/452	55.9%	66/118	0.000

Baseline characteristics of dysphagic patients without tube placement and those with tube insertion are compared in Table 2. Patients who required tube feeding had higher NIHSS scores (13.4±6.8 vs. 8.2±6.5; *p*<0.001) and were more often women (48.7% vs. 27.2%; *p* = 0.022). Age was not significantly different between groups (68.9±15.2 vs. 64.0±14.0; *p* = 0.091).

**Table 2:** Subgroup analysis of patients with dysphagia.

	Dysphagia without tube feeding (n = 81)		Dysphagia with tube feeding (n = 37)		*p*
Age	64.0±14.0		68.9±15.2		0.091
NIHSS at baseline	8.2±6.5		13.4±6.8		0.000
Male gender	72.8%	59/81	51.4%	19/37	0.022
Arterial hypertension	75.0%	60/80	75.0%	27/36	1.000
Hypercholesterolaemia	64.2%	52/81	56.8%	21/37	0.440
Diabetes mellitus	19.8%	16/81	24.3%	9/37	0.573
Smoking	38.4%	28/73	39.4%	13/33	0.919
Anterior stroke territory	79.0%	64/81	83.8%	31/37	0.624
Posterior stroke territory	18.5%	15/81	8.1%	3/37	0.176
Anterior and posterior stroke territory	2.5%	2/81	8.1%	3/37	0.177
Brainstem involvement	19.8%	16/81	10.8%	4/37	0.230
Thrombolysis	50.6%	41/81	67.6%	25/37	0.085

There were no major differences in the frequency of dysphagia stratified to stroke location: dysphagia was observed in 95 (21.8%) patients with anterior circulation stroke, 18 (17.3%) with posterior circulation stroke, and 5 (17.9%) with both anterior and posterior circulation stroke. Of 82 patients with brainstem infarction, 20 (24.4%) had dysphagia. The frequency of dysphagia at hospital discharge was similar in anterior, posterior and brainstem stroke (10.8%, 8.7%, and 13.4%, respectively). Dysphagia persisted in 4 of 5 patients with both anterior and posterior circulation stroke.

**Fig 1:**
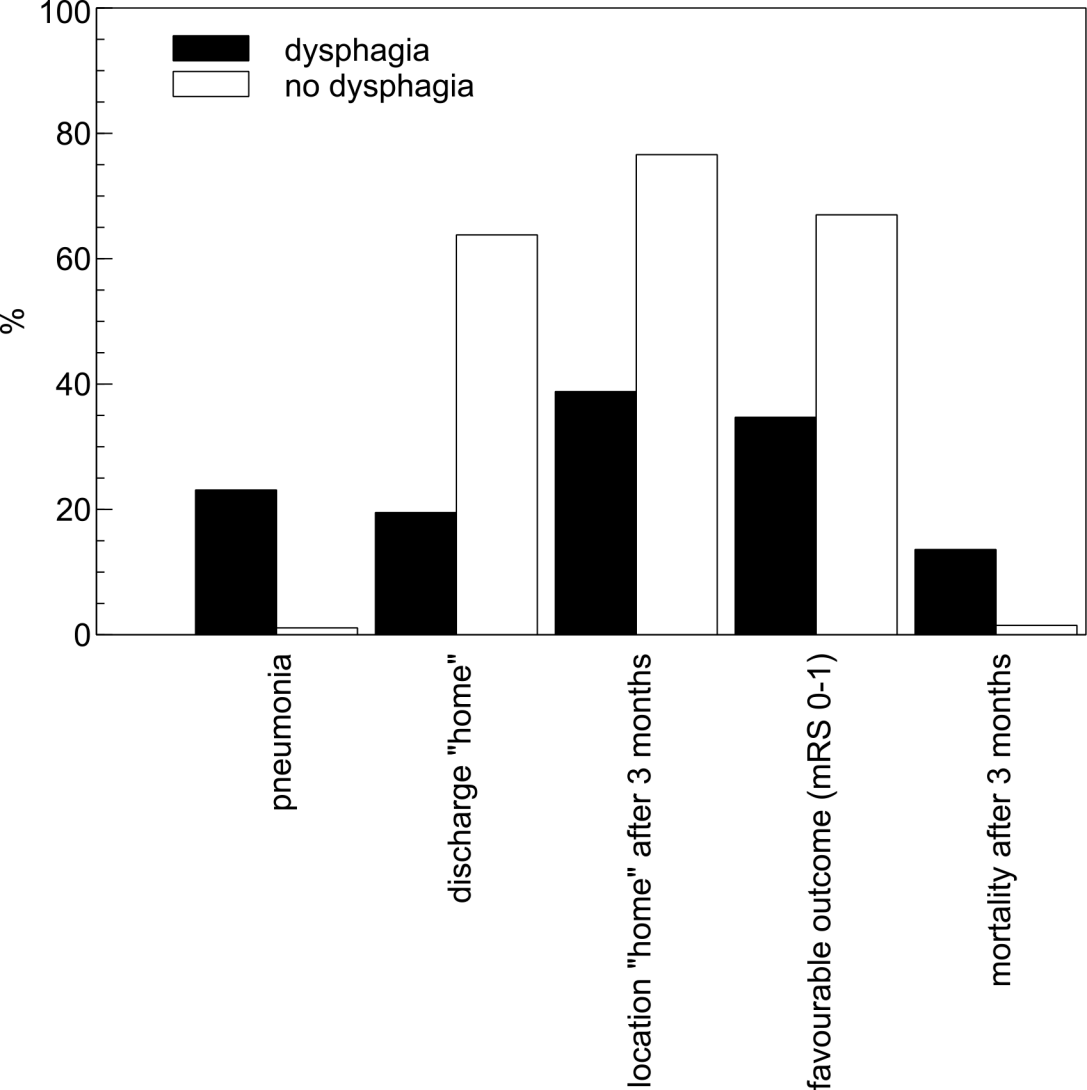
Comparison of outcome variables in dysphagic and non-dysphagic patients.

Tables 3 and 4 summarize the severity and persistence of dysphagia among stroke patients with swallowing dysfunction. During hospitalization, 36 of 118 (30.5%) patients with dysphagia needed a nasogastric tube and one patient required a percutaneous endoscopic gastrostomy. At hospital discharge, dysphagia persisted in 60 of 118 (50.9%) patients who had dysphagia at admission. Tube feeding was ongoing in 23 (19.5%) patients at discharge. In-hospital pneumonia occurred in 27 (22.9%) patients with dysphagia and in 5 (1.1%) without dysphagia (*p*<0.001, see Fig 1). Accordingly, the use of antibiotics was markedly higher in patients with dysphagia than those without dysphagia (28.0% vs. 4.6%, *p*<0.001). Multivariate logistic regression analysis showed that dysphagia (OR, 27.4; 95% CI, 10.2–73.7; *p*<0.001) was independently associated with pneumonia. Compared to dysphagic patients without tube insertion, those receiving tube feeding had much higher risk for in-hospital pneumonia, need of antibiotic treatment and death at 3 months (35.1% vs. 17.3%, *p* = 0.035, 51.4% vs. 17.3%, *p*<0.001, and 27.0% vs. 7.4%, *p* = 0.004, respectively). However, after adjustment for confounders, the association between tube placement and pneumonia was no longer statistically significant (OR, 2.2; 95% CI, 0.89–5.5; p = 0.087).

**Table 3:** Severity of dysphagia among 118 patients with swallowing disorder.

	%	n
Dysphagia requiring tube feeding	31.3	37/118
Dysphagia requiring nasogastric tube feeding	30.5	36/118
Dysphagia requiring PEG*	0.8	1/118

* PEG stays for percutaneous endoscopic gastrostomy

**Table 4:** Persistence of dysphagia among 118 patients with swallowing disorder.

	%	n
Persisting dysphagia at hospital discharge	50.9	60/118
Persisting dysphagia requiring tube feeding at discharge	19.5	23/118

Patients with dysphagia more often underwent chest radiographs (0.5±0.9 vs. 0.2±0.9 per person, *p* = 0.004) during hospital stay and were admitted to the stroke unit longer (4.4±2.8 vs. 2.7±2.4 days, *p*<0.001) as compared to patients without dysphagia. The total duration of hospital stay was similar in both groups (7.9±4.8 vs. 7.2±4.4 days, *p* = 0.145). Distinctive differences were observed with respect to discharge destination (Fig 1): patients with dysphagia had a higher probability for transfer to a rehabilitation clinic and a lower chance to be discharged home as compared to non-dysphagic patients (78.0% vs. 35.4%, *p* = 0.001 and 19.5% vs. 63.7%, *p* = 0.001, respectively). At 3 months, patients presenting with dysphagia were still less likely to live at home than patients with normal swallowing (38.8% vs. 76.5%, *p*<0.001).

In-hospital mortality was slightly higher in dysphagic patients (2.5% vs. 0.7%, *p* = 0.075), but at 3 months, mortality clearly increased in patients with dysphagia (13.6% vs. 1.6%, *p*<0.001). The frequency of favourable outcome was lower in dysphagic patients (35.6% vs. 69.7%, *p*<0.001) and half (50.0% vs. 21.4%, *p*<0.001) were living at a healthcare unit or rehabilitation clinic (Fig 1).

Multivariate logistic regression analyses identified stroke severity measured by NIHSS to be an independent predictor of dysphagia (odds ratio [OR], 1.1; 95% confidence interval [CI], 1.1–1.2; *p*<0.001). Discharge destination was significantly affected by dysphagia (OR, 4.7; 95% CI, 2.7–7.9; *p*<0.001) and stroke severity (OR, 1.1; 95% CI, 1.1–1.2; *p*<0.001).

Predictors of mortality at 3 months were severe dysphagia requiring tube feeding (OR, 8.5; 95% CI, 2.8–26.2; *p*<0.001), in-hospital pneumonia (OR, 9.7; 95% CI, 3.2–29.4; *p*<0.001), and brainstem involvement (OR, 4.1; 95% CI, 1.4–12.3; *p* = 0.011). Severe dysphagia requiring tube feeding, persisting dysphagia at hospital discharge and baseline stroke severity were inversely associated with favourable outcome at 3 months (OR, 0.3; 95% CI, 0.1–0.9; *p* = 0.028; OR, 0.2; 95% CI, 0.1–0.4; *p*<0.001; and OR, 0.96; 95% CI, 0.93–0.99; *p* = 0.016, respectively). The following factors were independently associated with institutionalization at 3 months: dysphagia (OR, 3.1; 95% CI, 1.7–5.5; *p*<0.001), stroke severity (OR, 1.2; 95% CI, 1.2–1.3; *p*<0.001) and brainstem involvement (OR, 0.3; 95% CI, 0.1–0.8; *p* = 0.008).

## Discussion

In this study, dysphagia affected more than one in five patients with ischemic stroke and was independently associated with in-hospital pneumonia, discharge destination and institutionalization, while severe dysphagia was a strong predictor of unfavourable outcome and mortality at 3 months. Furthermore, dysphagia had a significant impact on increased healthcare consumptions such as chest radiographs or antibiotics.

The reported frequency of dysphagia after stroke varied considerably in earlier studies, ranging from 50% to 80% [1,17–20]. In our study, the incidence of dysphagia was substantially lower than these estimates. These variations can be attributed to differences in definition and assessment of dysphagia, timing of swallowing examination, and patient selection. Advances in acute stroke treatment and management at a stroke unit may also have contributed to the lower rate in this study. Nevertheless, the frequency of swallowing dysfunction in our study still accounts for a substantial proportion, thus a regular and precise screening in patients with acute stroke is advisable.

An important finding of this study is the large impact of dysphagia on clinical outcome at 3 months. Patients with dysphagia had an 8.5-fold higher risk of death as compared to those with normal swallowing. Furthermore, dysphagia was independently associated with institutionalization of stroke patients at 3 months. In addition, multivariate analyses showed tube placement due to severe dysphagia was an independent predictor of mortality and unfavourable outcome. These observations are in line with other studies reporting increased mortality, less favourable outcome and higher institutionalization rates at 3 months in stroke patients presenting with dysphagia [3,4]. Stroke severity was another significant determinant for clinical recovery and residency at 3 months. Of note, mortality at 3 months was associated more strongly with severe dysphagia than with stroke severity at baseline. These data indicate that dysphagia has a large effect on survival, clinical recovery, and dependency after stroke. The use of thrombolysis was higher in patients presenting with dysphagia. This probably relates to the fact that dysphagia is associated with severe strokes and that thrombolysis is used more often in severe cases. Similar findings were reported by Okubo and colleagues [21].

It is well known that stroke is associated with high in-hospital costs and our study indicates that dysphagia may additionally increase the medical expenses. Affected patients more often underwent chest radiographs and antibiotic treatment, stayed longer at a stroke unit and more frequently were transferred to rehabilitation clinics. On the other hand, the overall duration of hospital stay was not significantly different in patients with normal swallowing compared to those with dysphagia, which contradicts results from earlier studies [22–24]. This difference may be explained by our policy to transfer stable stroke patients at an early stage to primary care hospitals in the Bernese stroke network. However, dysphagic patients stayed significantly longer at stroke unit as compared to those without dysphagia. A recent study from South Carolina reported that dysphagia after stroke significantly increases medical costs, driven by higher hospital and durable medical equipment expenses [24]. The 1-year costs of post-stroke dysphagia was estimated to be $4’510, as compared to $1’703 attributable to post-stroke aphasia [24,25].

Patients with dysphagia had a much higher risk for pneumonia as compared to patients with normal swallowing in our study, which may further increase the hospitalization costs [26]. This finding is consistent with a review that indicated a 3-fold increased risk of pneumonia in patients with dysphagia and an 11-fold increased risk in those with aspiration [1]. Furthermore, pneumonia predicted increased risk of death at 3 months. This observation is in line with a large multi-center study reporting an increased 30-day and 1-year mortality due to stroke-associated pneumonia [27]. The incidences of post-stroke pneumonia in intensive care units range from 22% to 47%, depending on study design and definition of chest infection [28,29]. In comparison, the overall rate of pneumonia in our study population was rather low (6%). Regular dysphagia screening in stroke units by trained therapists may explain this, as this strategy has been shown to decrease the incidence of pneumonia in acute stroke patients [30,31]. On the other hand, still one in four stroke patients with dysphagia developed pneumonia despite regular monitoring. This observation calls for further improvement in the management of dysphagic patients. Recent studies indicate that functional magnetic or electrical stimulation may improve dysphagia as compared to conventional swallowing therapy, but these results need to be confirmed by larger studies [32,33]. There could be a need for such novel approaches, as swallowing dysfunction may persist in many patients for a longer time [34,35]. In our study, half of all patients with initial dysphagia still had dysphagia at hospital discharge.

Our study indicates that initial stroke severity is the main risk factor for dysphagia as shown by previous studies [3,21]. Stroke location was not associated with dysphagia in our study. This finding contradicts the assumption that dysphagia may be mainly caused by posterior circulation or brainstem strokes. Accordingly, studies have shown that total anterior circulation strokes have the highest frequency of dysphagia and lesions of the frontal and insular cortex independently predict prolonged dysphagia after stroke [3,22,36,37]. Supratentorial strokes such as lesions of pre-motor or motor cortices and basal ganglia may cause dysphagia by affecting the planning and execution of swallowing or pharyngeal peristalsis, whereas brainstem lesions may impair oro-pharyngeal sensation, laryngeal elevation and timing of pharyngeal swallow [2,38]. Rates of recovery from dysphagia were comparable in anterior and posterior circulation stroke in our study. Furthermore, simultaneous lesions in anterior and posterior circulation stroke were associated with an unfavourable prognosis, but the low number of patients prohibits any firm conclusions. Similar to our findings, stroke territory did not influence the prognosis of dysphagia in other studies [17,36]. Poor recovery has been reported in severe strokes and especially large lesions of primary motor cortex, whereas dysphagia after brainstem stroke had a favourable outcome on long-term [20,22,39]. Patients with brainstem infarction were at lower risk for institutionalization in our study. We believe that this may be mainly associated with the severity of stroke, which is supposed to be less severe in brainstem involvement. Accordingly, several studies have shown that patients with posterior circulation stroke have lower National Institutes of Health Stroke Scale (NIHSS) scores than patients with anterior circulation stroke [40–42]. In addition, large infarcts in territory of middle cerebral artery (MCA) often cause severe hemiparesis, cognitive impairment, aphasia or neglect requirung intense rehabilitation and / or institutionalization. In contrast, in brainstem infarctions higher cortical functions are not affected and many patients with brainstem infarct and without pyramidal tract involvement do not have paresis (e. g. Wallenberg’s syndrome). Therefore, patients with brainstem infarctions may be at lower risk for institutionalization at 3 months. Finally, selection bias may be another cause for this observation: patients with severe brainstem strokes generally have markedly higher mortality rates and we may have followed just survivors with small brainstem infarcts at 3 months, whereas hemicraniectomy in severe MCA stroke often decrease mortality without improving clinical recovery (survivor bias).

Another finding of our study is that patients with nasogastric tube placement had a much higher risk of death as compared to dysphagic patients without tube insertion, whereas the association with the occurrence of pneumonia was rather weak in multivariate analyses. Langdon and colleagues reported significantly higher rates of respiratory infection in strictly tube-fed stroke patients as compared to those fed orally [43]. On the one hand, one may argue that tube placement is probably a marker of increased aspiration risk as this was performed in patients with large infarcts and severe dysphagia (mainly GUSS score <10 points). However, it is still under debate whether placement of nasogastric tubes additionally increases the risk of pneumonia by promoting colonization of oropharynx with pathogenic bacteria [44,45], as most aspiration pneumonia are assumed to result from bacterial origin [46]. Noteworthy, feeding tubes do not prevent aspiration of gastroesophageal reflux [44,47]. A retrospective review indicated nasogastric tubes and immobility to be stronger predictors for respiratory infections than dysphagia in acute stroke [48]. These data suggest that the benefit and harm of nasogastric tubes needs to be investigated in further studies. Stringent oral hygiene, attention to upright positioning during enteral feeding, mobilization of bedridden patients and routine change of nasogastric tubes may decrease the risk of pneumonia [43,49–51].

This study has several limitations. The retrospective study design is the main drawback, although dysphagia, baseline data, risk factors and clinical outcomes were assessed routinely in patients. GUSS has been shown to be a reliable method for dysphagia screening, however, no consensus exists on the optimal diagnostic tool [52]. Another limitation is the use of NIHSS score as the only marker of stroke severity, whereas volume measurements of infarct lesions were not performed due to variations in types of imaging. Furthermore, classification of dysphagia was rather broad as it was dichotomized between dysphagia and severe dysphagia only. In addition, we did not have data on pre-existing comorbidity that may have resulted in swallowing impairment prior to stroke. In view of broad confidence intervals, the results must be interpreted cautiously and need to be confirmed by larger studies. Finally, our findings may not be generalized with respect to our monocentric study design in a tertiary care stroke center.

In summary, this study indicates that dysphagia still affects a substantial portion of stroke patients. Dysphagia was clearly associated with clinical outcome, mortality and healthcare expenses despite advanced stroke treatment. In view of this burden, our data call for further research to improve management of dysphagia in acute stroke.
